# Management of Midline Diastema in a Young Adult With Minimal-Thickness Porcelain Laminate Veneers

**DOI:** 10.7759/cureus.41904

**Published:** 2023-07-14

**Authors:** Sheetal Vijaya, Shilpa Vijaya, Meghan J Shetty

**Affiliations:** 1 Department of Prosthodontics & Crown and Bridge, Implantology, Srinivas Institute of Dental Sciences, Mangalore, IND; 2 General Dentistry, Private Practice, Glen Allen, USA; 3 Department of Prosthodontics & Crown and Bridge, Implantology, A.J. Institute of Dental Sciences, Mangalore, IND

**Keywords:** aesthetics, lithium disilicate, ips e.max, diastema, dental veneers

## Abstract

Treating healthy but unaesthetic teeth has been a major challenge for a dental practitioner. Maxillary midline diastemas are one such aberration in the smile zone. Although a diastema can be transient in the developing dentition, the management of the diastema in adulthood requires a detailed examination and appropriate care for a successful outcome. Majority of specialty dentists prefer conservative or minimally invasive treatments, such as porcelain laminate veneer (PLV) restorations over full-ceramic crowns for anterior teeth, where esthetics can be extremely concerning. Minimal-thickness laminate veneers provide a satisfactory outcome while preserving the dental structure as a whole. Thus, this clinical report describes a conservative approach of minimal preparation utilizing a restoration to accomplish both esthetic and functional rehabilitation.

## Introduction

The appearance of a diastema in the anterior region is esthetically challenging for dental practitioners as it distorts a pleasing smile [1-5]. Maxillary midline diastemas (MMDs) can be defined as a space larger than 0.5 mm between the proximal surfaces of the two central incisors [1]. Although various treatment options have been documented for addressing midline diastemas, achieving a satisfactory and durable esthetic outcome relies on a thorough diagnosis and meticulous treatment planning.

The idea of providing minimally invasive treatment and understanding the options for the preservation of teeth are some of the important aspects of prognosis [6]. Veneers are considered a reliable restorative procedure due to their color stability, strength, biocompatibility, most conservative, excellent clinical performance, and high esthetic outcome [7-9].

This clinical report presents a conservative and minimally invasive approach to close a midline diastema using lithium disilicate veneers.

## Case presentation

A 22-year-old young man reported to the Department of Prosthodontics at a private dental institute with a complaint of spacing between his upper front teeth and desired long-term correction with a primary focus on improved esthetics. Although there was no history contributing to the existing diastema, it was noted that the patient had a dental history of using orthodontic retainers in an attempt to close the space. All the routine clinical and radiographic investigations were well within normal limits.

**Figure 1:**
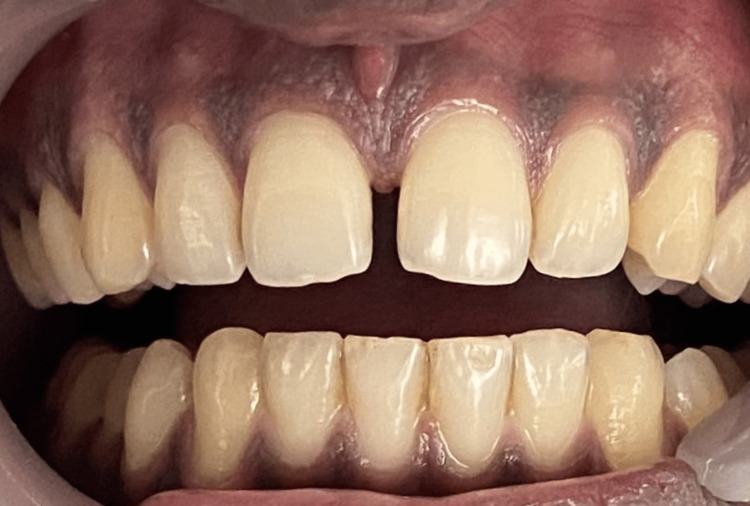
Pre-operative frontal view of the midline diastema.

On intra-oral examination, a diastema of 3 mm with respect to the maxillary right and left central incisors (Figure 1) was observed with an overjet and an overbite of 2 and 3 mm, respectively. The patient presented with a sound periodontal condition, thin gingival biotype, good oral hygiene, and a medium frenal attachment (Class II). In addition to these findings, a spacing of 1 mm was observed between the maxillary left lateral incisor and canine, and a dental midline shift was noted. However, the patient deferred treatment with respect to the midline shift and prioritized getting treatment for the correction of the MMD. The treatment options given to the patient were orthodontic closure of space, direct restoration using composite resin material, and porcelain laminate veneers (PLV). The advantages and disadvantages of each treatment were explained, and the patient opted for the PLV due to its conservative approach, longevity, and short duration of treatment. Informed consent was taken prior to treatment that explained the benefits, drawbacks, and complications associated with the treatment. 

**Figure 2:**
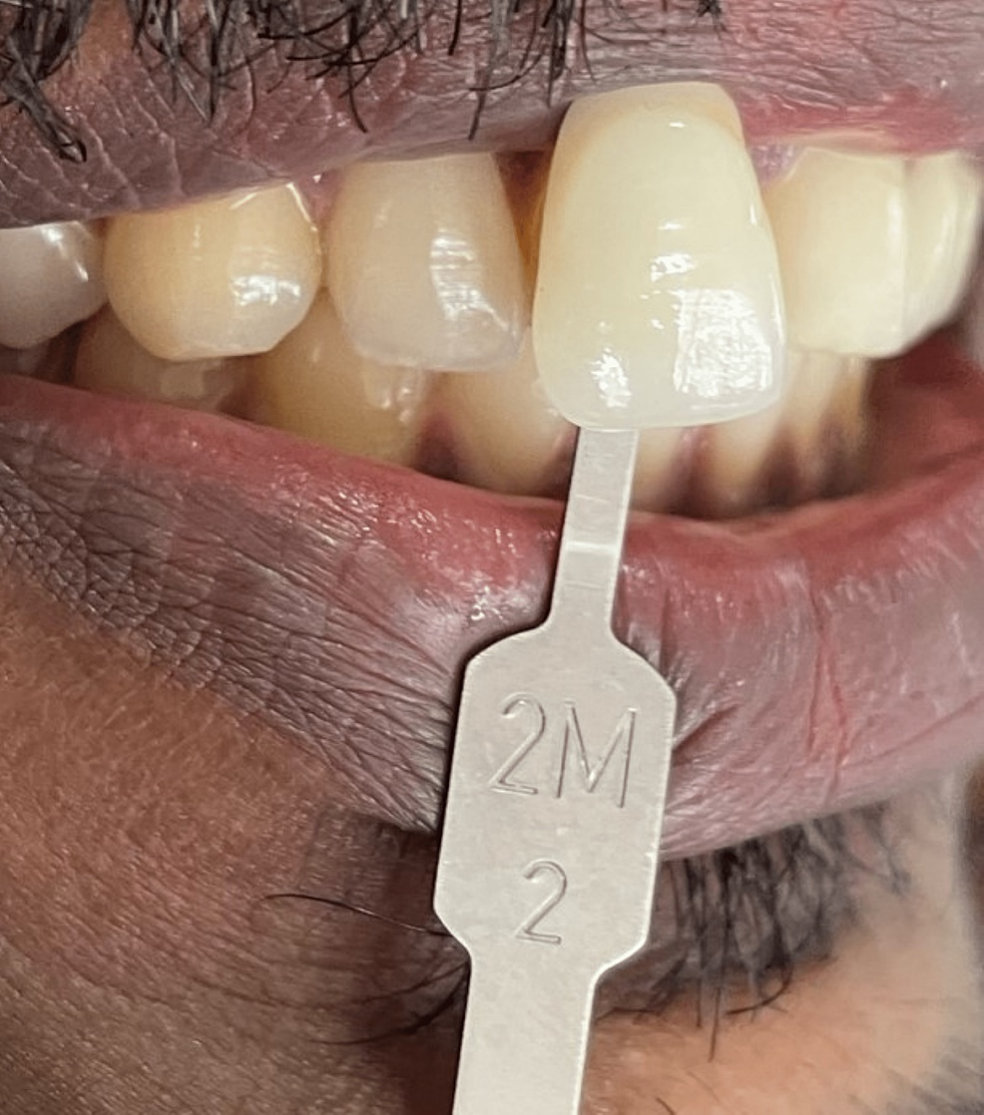
Shade selection.

**Figure 3:**
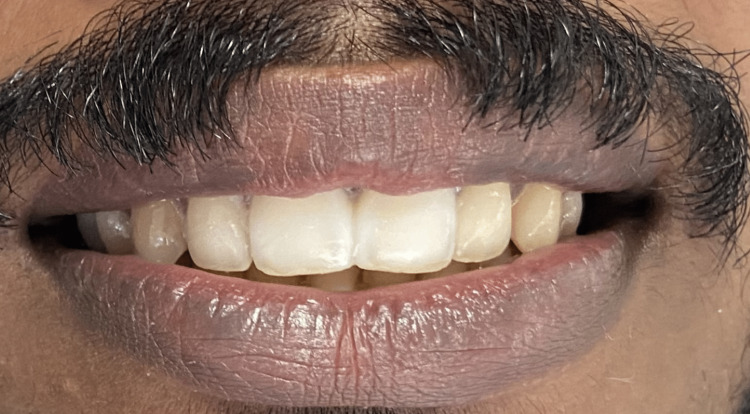
Esthetic trial performed to evaluate the prosthetic outcome.

The diagnostic impressions were made using irreversible hydrocolloid (BioKalgin Pro®, Brulon, India) and poured with Type III dental stone (Goldstone®, India), and the study casts were retrieved for treatment planning. One set of study casts was used for the diagnostic wax-up of the maxillary central incisors and in the second set of study casts. Mock preparations were made in relation to the maxillary central incisors. Shade selection was done using VITA Toothguide 3D-MASTER® (Vita Zahnfabrik, Germany) (Figure 2). A test run was carried out using bis-acrylic composite (Protemp 4, 3M ESPE, USA), which allowed the patient to visualize a trial smile before the preparations were initiated (Figure 3). 

**Figure 4:**
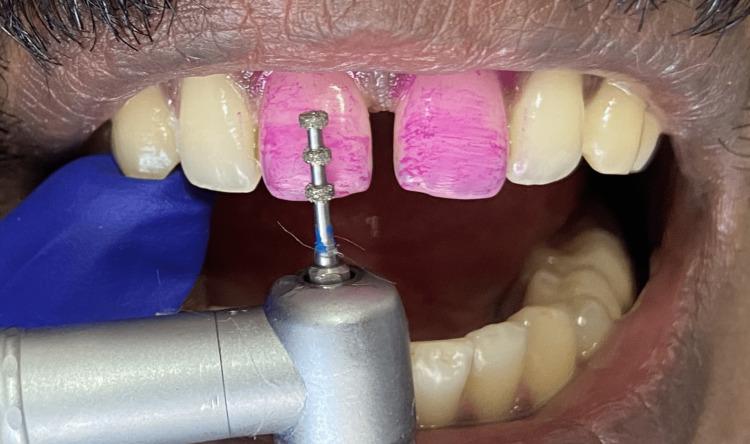
Placement of depth orientation grooves using LSV1 bur.

**Figure 5:**
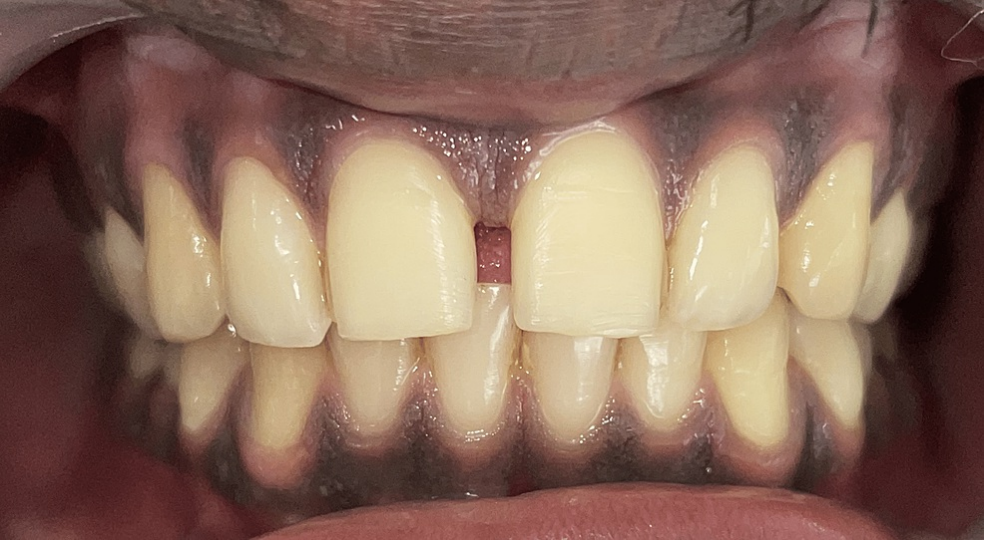
Minimally invasive preparations done with respect to maxillary central incisors (Incisal overlap with proximal wrap-around).

The preparation design decided was incisal overlap with proximal wrap-around in relation to the maxillary right and left central incisors. The preparations began with the placement of initial depth orientation grooves on the labial surface using LVS1 bur (Figure 4). Labial reduction of 0.5 mm was done using a long, round-end tapered standard grit diamond bur to obtain a chamfer finish line with 0.2-0.3 mm sulcular extension. The distal finish line was terminated 0.2 mm labial to the contact area while the mesial finish line was extended palatally due to the presence of diastema. Incisal reduction of 1 mm, sloping gingivally from the labial, was done. Lingual enamel was modified to form a rounded chamfer to a depth of 0.5-1 mm. The overall reduction was confined to the enamel to ensure superior bonding and avoid postoperative sensitivity, which may develop if the dentino-enamel junction was involved in the preparation (Figure 5).

**Figure 6:**
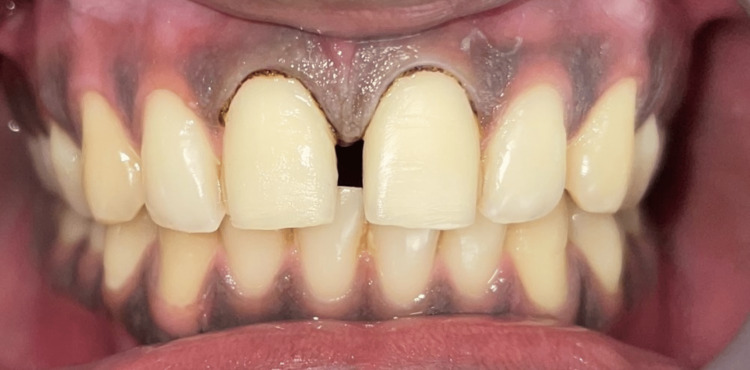
Gingival retraction using #00 retraction cords in hemostatic gel.

**Figure 7:**
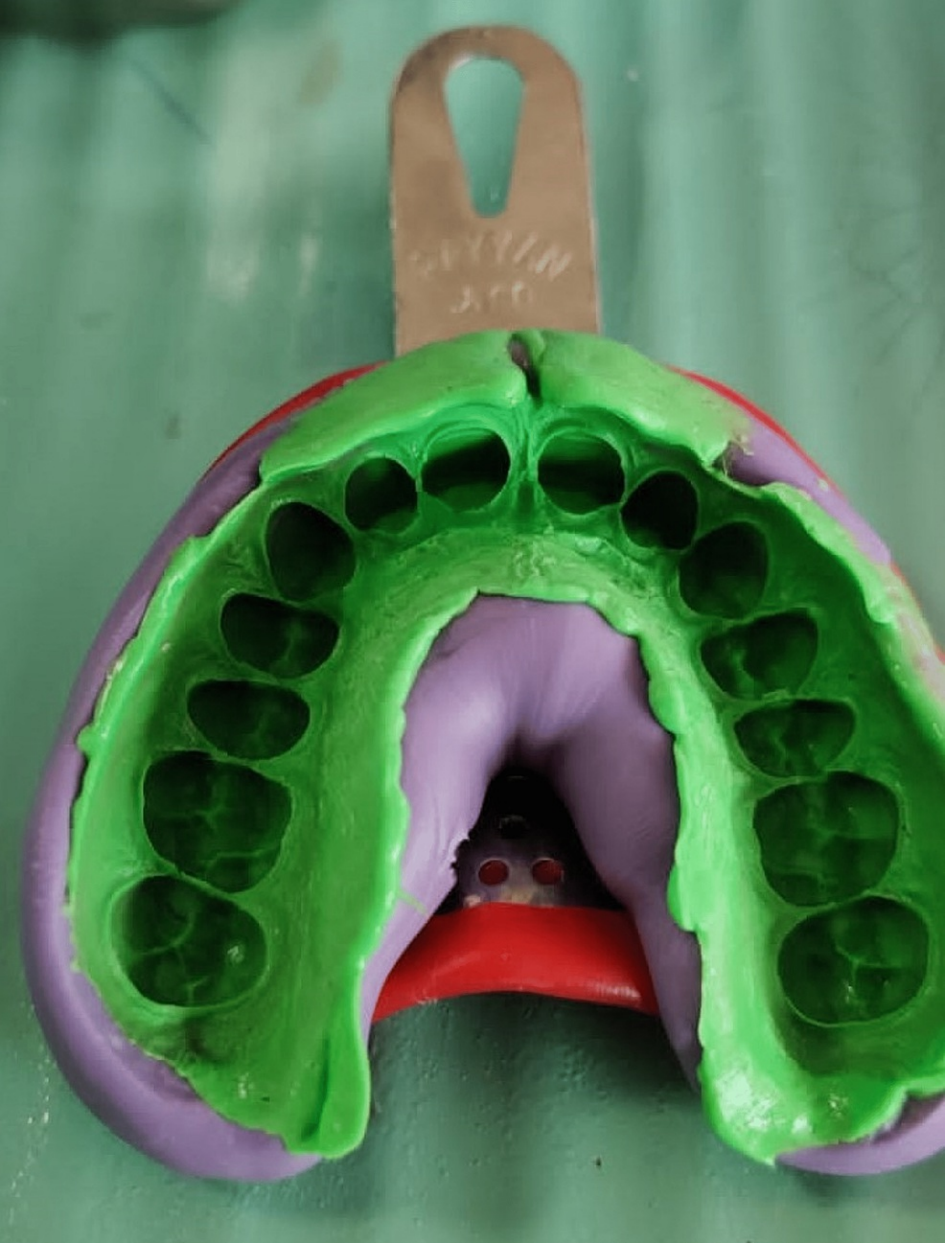
Putty-wash impression using polyvinyl siloxane material in a metal tray.

Gingival retraction was achieved using a gingival retraction cord (Primecord #00, Prime Dental, India) impregnated with hemostatic gel (Hemostal Gel™, Prevest DenPro, USA) for two minutes (Figure 6). Final impressions were made with a two-stage putty wash technique using vinyl polysiloxane material (Photosil™, DPI, India) in a custom tray and sent to the laboratory (Figure 7). No provisionals were made since the preparation was restricted to the enamel. A layer of bonding agent (Single Bond Universal Adhesive, 3M ESPE, Germany) was applied on the enamel as a protective coating. 

**Figure 8:**
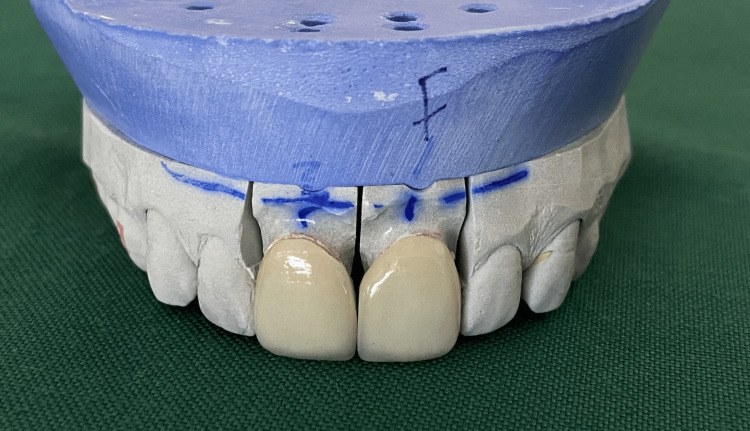
Veneers fabricated on the die stone model.

Laminate veneers were fabricated with lithium disilicate material (IPS e.max Press, Ivoclar Vivadent, Liechtenstein) using the heat press technique. A layering ceramic (IPS e.max Ceram, Ivoclar Vivadent) was further applied to promote the incisal edge optical characteristics (Figure 8).

A day later, clinical try-in of the laminate veneers was done to check the marginal fit, contour, color match, contacts, and protrusive interferences after receiving them from the laboratory. Veneers were deemed acceptable upon satisfactory clinical seating without occlusal interferences on the lingual and incisal aspects. Preoperative photographs were taken for documentation.

**Figure 9:**
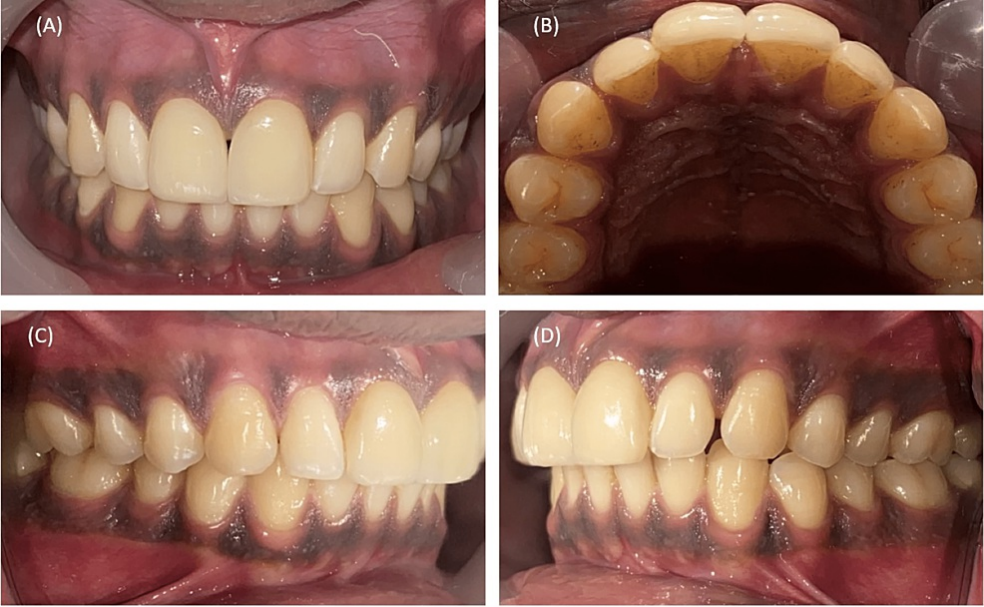
Completed restoration after final cementation. A, Frontal view. B, Occlusal view. C, Right lateral view. D, Left lateral view.

Two days following the try-in, on the day of cementation, the intaglio surface of the veneers was etched with Ivoclean or 5% hydrofluoric acid (IPS Ceramic Etching Gel, Ivoclar Vivadent AG) for 20 seconds, rinsed and dried followed by the application of silane coupling agent (Monobond® N, Ivoclar Vivadent). The prepared tooth surfaces were rinsed with pumice slurry and etched with 35% phosphoric acid (Scotchbond™, 3M ESPE) for 15 seconds, rinsed with water and air dried. This was followed by the application of a bonding agent (Single Bond Universal Adhesive, 3M ESPE). Light cure resin luting agent of translucent shade (RelyX™ Veneer cement, 3M ESPE) was placed on the intaglio surface of the porcelain veneers and placed gently on the teeth using an adhesive tip applicator. Margins were inspected for complete seating and tack cured for five seconds. Excess luting cement was removed followed by light curing for 30 seconds on each tooth for complete polymerization. Veneers were examined for occlusal interferences (Figure 9). Contacts were verified with floss and were found to be acceptable. 

**Figure 10:**
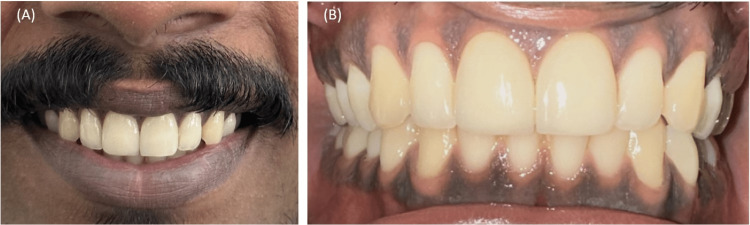
Clinical evaluation of veneers 12 months postoperatively with desirable esthetics.

Following the seating of veneers, a significant improvement in esthetics was observed. In addition, the patient was recommended to maintain good oral hygiene, avoid using the front teeth to directly bite into food to preserve the longevity of the restoration and was scheduled for one week, one month, six months, and a year recall visit (Figure 10). 

## Discussion

Certain aberrations, such as spacing or midline diastemas in the smile zone, are common, resulting in an esthetic concern among adults. MMDs are a major concern for most of the patients; therefore, determining the etiologic factors is crucial [1-3]. Among the factors included are labial frenum attachment, discrepancy in tooth size or shape, congenitally missing lateral incisors, and tongue or lip habits. While a great majority of diastemas close after the eruption of maxillary canines, patients with diastemas greater than 2 mm and generalized spacing are at risk of not closing with normal development [3,4]. Several treatment options employed to close the midline diastemas due to tooth-size discrepancy include direct restorations using composite resins, indirect restorations such as PLV, full contour porcelain crowns, and orthodontic treatment. 

Diastemas of 1-1.5 mm can be closed using direct restorations, such as composite resin. Although composite restorative material is easy to handle and economical and requires few appointments, it has the disadvantage of being less wear-resistant and gathers stains over a period of time. In addition, orthodontic treatment is frequently denied by young adults because of its long-term duration with multiple appointments to enhance their smile [1,4]. A conservative, quick, and long-lasting alternative to composite resin with excellent tissue response and minimal preparation is PLV.

Laminate veneers are extensively utilized to achieve precise proximal contacts with adequate overbite and overjet and hence are regarded as a more conservative option compared to ceramic crowns [4]. An increasing desire for esthetically appearing dental restoration has led to the advancement of various dental ceramics. Glass ceramics have optical characteristics similar to that of the tooth, are highly accurate, and have minimal internal structural defects and good bonding to the enamel, making them more resilient after cementation [6,7]. However, some of their limitations include large Class IV defects and inadequate remaining enamel for retention of the veneer. Calamia and Simonsen demonstrated that the bond strength of hydrofluoric acid-etched and silanized veneer to the luting resin composite is generally greater than the bond strength of the same luting resin to the etched enamel surface [7,10]. 

Morimoto et al. conducted a systematic review that involved identifying 899 studies, out of which only 13 were analyzed. The findings of the review revealed an overall survival rate of 89% at nine years [11]. Interestingly, the survival rate for glass-ceramic veneers (94%) was higher compared to that for feldspathic porcelain veneers (87%). Therefore, for the long-term survival of PLV, several key factors contribute to success. These factors include careful case selection, comprehensive treatment planning, preparations terminating in enamel, appropriate material selection, suitable techniques, and collaboration with a skilled ceramist.

There has been a paradigm shift in contemporary prosthodontics to assist the emerging interest in implementing conservative restorative choices. In the recent literature, there has been an increased focus and demand on the delivery of minimally invasive dentistry to provide optimal care with minimal removal of tooth structure. Hence, it has been regarded as a feasible approach to maintain the patient’s tooth structure to enhance good bonding, retention, esthetics, and strength.

## Conclusions

Various techniques, such as composite resin restoration, PLV, full-contour porcelain crowns, and orthodontic treatment, have been used in the literature to treat midline diastemas. Although the composite restorative materials are cost-effective and less time-consuming, they have a main drawback of getting stained over time and require frequent follow-ups. Alternatively, laminate veneers are a conservative option requiring no or minimal enamel preparation. In addition, they offer excellent esthetic outcome when done in a minimally invasive approach. This clinical report demonstrates a conservative enamel preparation design to manage diastemas using minimal-thickness PLV without compromising the esthetics and function. Furthermore, the evolution of restorative materials along with advances in adhesive technology facilitates clinicians to deliver esthetic restorations.
